# Association between Enzyme-Linked Immunosorbent Assay-Measured Kidney Injury Markers and Urinary Cadmium Levels in Chronic Kidney Disease

**DOI:** 10.3390/jcm11010156

**Published:** 2021-12-29

**Authors:** Kai-Fan Tsai, Pai-Chin Hsu, Chien-Te Lee, Chia-Te Kung, Yi-Chin Chang, Lung-Ming Fu, Yu-Che Ou, Kuo-Chung Lan, Tzung-Hai Yen, Wen-Chin Lee

**Affiliations:** 1 Division of Nephrology, Department of Internal Medicine, Kaohsiung Chang Gung Memorial Hospital, Chang Gung University College of Medicine, Kaohsiung 83301, Taiwan; b9302095@cgmh.org.tw (K.-F.T.); nick9335@cgmh.org.tw (P.-C.H.); ctlee33@cgmh.org.tw (C.-T.L.); 2 Department of Emergency Medicine, Kaohsiung Chang Gung Memorial Hospital, Chang Gung University College of Medicine, Kaohsiung 83301, Taiwan; g00308@cgmh.org.tw; 3 Division of Infectious Diseases, Department of Internal Medicine, Kaohsiung Chang Gung Memorial Hospital, Chang Gung University College of Medicine, Kaohsiung 83301, Taiwan; b9502045@cgmh.org.tw; 4 Department of Engineering Science, National Cheng Kung University, Tainan 701401, Taiwan; loudyfu@mail.ncku.edu.tw; 5 Department of Obstetrics and Gynecology, Kaohsiung Chang Gung Memorial Hospital, Chang Gung University College of Medicine, Kaohsiung 83301, Taiwan; Tedcou@gmail.com (Y.-C.O.); blue@cgmh.org.tw (K.-C.L.); 6 Clinical Poison Center, Department of Nephrology, Chang Gung Memorial Hospital, Taoyuan 333423, Taiwan; m19570@cgmh.org.tw

**Keywords:** cadmium, chronic kidney disease (CKD), enzyme-linked immunosorbent assay (ELISA), kidney injury molecule-1 (KIM-1)

## Abstract

Cadmium exposure is associated with chronic kidney disease (CKD), but the optimal biomarker for early cadmium-associated nephrotoxicity in low-level exposure has not yet been established. We conducted a cross-sectional investigation involving 167 CKD patients stratified according to tertiles of urinary cadmium levels (UCd), in which enzyme-linked immunosorbent assay (ELISA)-measured novel renal biomarkers were utilized to assess the extent of renal injury associated with cadmium burden. In the analyses, urinary kidney injury molecule-1 (KIM-1) levels and age were the independent factors positively correlated with UCd after adjusting for covariates in non-dialysis-dependent CKD patients (high vs. low UCd, odds ratio (95% confidence interval), 1.0016 (1.0001–1.0032), *p* = 0.043, and 1.0534 (1.0091–1.0997), *p* = 0.018). Other conventional and novel renal biomarkers, such as serum creatinine, estimated glomerular filtration rate, CKD staging, urinary protein/creatinine ratio, urinary 8-hydroxy-2-deoxyguanosine (8-OHdG), and urinary epidermal growth factor (EGF) were not independently correlated with UCd in the analyses. In conclusion, our study found that the ELISA-measured urinary KIM-1 level could serve as an early renal injury marker in low-level cadmium exposure for non-dialysis-dependent CKD patients. In addition, age was an independent factor positively associated with UCd in this population.

## 1. Introduction

Cadmium is a toxic metal element that exerts several adverse effects on human health, including bone disease, cardiovascular disease, infertility, malignancy, and chronic kidney disease (CKD) [1]. In addition to high-level exposure from occupational contact or pollution, low-level cadmium exposure via daily environmental sources is also associated with human diseases, such as hypertension, dyslipidemia, lung disease, and renal disease [2,3,4,5,6]. With a half-life of up to 30 years, cadmium accumulates for a long time in several organs such as the kidney, liver, tooth, and bone, and it persistently affects human health [7,8]. Compared to the Western population, individuals from South Asia and East Asia are believed to have a higher possibility of environmental cadmium exposure, which is mainly derived from cigarette and dietary sources; hence, cadmium exposure is an important issue in this population [9,10]. Among the multiple health effects of cadmium, its nephrotoxicity is well known, with an association between low-level environmental cadmium exposure and risk for CKD found in the literature [11,12]. Mechanisms of cadmium-induced nephrotoxicity include tubular injury, especially in the proximal tubule, intracellular oxidative stress, interstitial fibrosis, and reduction in the number of viable nephrons [1,13]. In previous reports conducted in Taiwan, blood and urinary cadmium concentrations, which are commonly used surrogates of body cadmium burden [14,15], were also positively correlated with proteinuria and a risk for CKD [16,17,18]. Considering the high prevalence of CKD and end-stage renal disease, which reached up to 15% and 3587 per million population in 2018, respectively, the influence of environmental cadmium exposure on CKD is a topic of concern in Taiwan [19,20]. Although the association between cadmium exposure and renal disease has been widely supported in the literature, an optimal biomarker for cadmium-induced nephrotoxicity has not yet been established. Despite the inverse relationship between estimated glomerular filtration rate (eGFR) and urinary cadmium concentration recognized in some studies, other studies indicated that creatinine-based eGFR might be paradoxically elevated with higher urinary cadmium [21,22,23]. Possible explanations for this paradox include the time lag between cadmium-induced cellular injury and absolute loss of functional nephrons, as well as the dependence of cadmium clearance on renal function, highlighting the role of novel renal biomarkers in the assessment of early cadmium-induced nephrotoxicity, especially in low-level chronic exposure [13,24].

Novel renal biomarkers, including tubular injury markers such as neutrophil gelatinase-associated lipocalin (NGAL), retinol-binding protein (RBP), kidney injury molecule-1 (KIM-1), β2-microglobulin (β2MG), and N-acetyl-d-glucosaminidase (NAG); oxidative stress markers such as 8-hydroxy-2-deoxyguanosine (8-OHdG); and fibrosis markers such as epidermal growth factor (EGF), have been utilized in various studies of kidney injury [25,26,27,28]. Currently, several commercial enzyme-linked immunosorbent assay (ELISA)-based tools are available to detect the above novel renal biomarkers in urine samples, making their clinical and research utilization feasible. In the general population, positive correlations between cadmium exposure and tubular injury markers, such as β2MG, NAG, and KIM-1, have been recognized in the literature, supporting their roles in the early detection of cadmium-induced nephrotoxicity [29,30]. In addition, urinary 8-OHdG levels were positively associated with urinary cadmium concentrations in a study of residents in industrial regions, reflecting increased oxidative stress related to cadmium exposure [31]. Altogether, traditional renal function surrogates such as eGFR might not be sensitive enough to detect cadmium-induced nephrotoxicity in the early phase and in the setting of low-level cadmium exposure, and ELISA-measured novel renal biomarkers emerge as convenient and reasonable tools in these situations. However, the clinical efficacy of ELISA-measured novel renal biomarkers in the evaluation of cadmium-induced renal injury has not been specifically studied in the CKD population. 

In this cross-sectional investigation, we assessed urinary cadmium concentrations in non-dialysis-dependent CKD patients and utilized ELISA-measured renal biomarkers, including urinary KIM-1, 8-OHdG, and EGF, to evaluate cadmium-associated renal injury in this population.

## 2. Materials and Methods

### 2.1. Patients

Patients living in geographically different regions in southern Taiwan were recruited from various outpatient clinics of Kaohsiung Chang Gung Memorial Hospital. They were referred to a multidisciplinary renal care program between January 2021 and June 2021. The inclusion criteria were as follows: (1) adult patients (≥20 years of age) with non-dialysis-dependent CKD stage 3a–5, and (2) patients receiving follow-up treatment for at least one year in the nephrology outpatient department of the hospital. Patients with a history of occupational or accidental heavy metal poisoning, liver cirrhosis, active infectious disease, malignancy under chemotherapy or radiotherapy, active alcoholism, or drug abuse were excluded. Pregnant patients and those who were hospitalized or underwent surgery within 3 months of enrollment were also excluded. This study protocol was approved by the Institutional Review Board and Ethics Committee of Chang Gung Medical Foundation, Taipei, Taiwan (IRB No. 202001027B0), and it adhered to the principles of the Declaration of Helsinki and Declaration of Istanbul. All patients provided informed consent.

### 2.2. Demographic Profiles and Clinical Characteristics Collection

The demographic profiles and clinical characteristics of the enrolled patients were collected at the enrollment visit and from the electronic medical record system of the hospital, including age, sex, body mass index (BMI), address of current residence, CKD staging, habits of smoking, drinking, and betelnut usage, as well as comorbidities, such as hypertension, diabetes mellitus, dyslipidemia, vascular disease, heart failure, chronic obstructive pulmonary disease (COPD), gout or hyperuricemia, history of malignancy, and transplantation history. Long-term medications continuously prescribed for at least 3 months, including antihypertensives, glucose-lowering agents (oral or injection), and lipid-lowering agents (such as statins, fibrates, and other related agents), were also recorded. Current residential address was utilized to determine whether the patient lived in an urban or rural area. The stage of CKD was defined according to the Kidney Disease Improving Global Outcomes 2012 Clinical Practice Guideline for the Evaluation and Management of CKD [32], and it was based on two consecutive serum creatinine (SCr) measurements (one at the enrollment visit and another within 3 months prior to enrollment) and the corresponding SCr-based eGFR levels. Considering the long half-life of cadmium, habits of drinking, smoking, and betelnut usage were recorded according to consumption history within 10 years prior to enrollment. Hypertension was defined as the regular use of at least one antihypertensive agent or having at least two blood pressure measurements above 140/90 mmHg. Diabetes was defined as regular use of at least one glucose-lowering agent or having at least two consecutive diagnostic tests with abnormal results (i.e., glycated hemoglobin (HbA1c) ≥ 6.5%, 8-h fasting blood glucose ≥ 6.99 mmol/L, or 2-h postprandial blood glucose ≥ 11.10 mmol/L). Dyslipidemia was defined as regular use of at least one lipid-lowering agent or having at least 2 consecutive tests revealing abnormal lipid profiles (i.e., total cholesterol ≥ 5.18 mmol/L, low-density lipoprotein cholesterol ≥ 3.37 mmol/L, high-density lipoprotein cholesterol ≤ 1.04 mmol/L, or triglyceride ≥ 1.69 mmol/L). Vascular disease, including cardiovascular disease, cerebrovascular disease, carotid artery disease, peripheral vascular disease, and other comorbidities, were extracted from the medical records. 

### 2.3. Measurement of Blood Biochemical Profiles and Urinary Renal Biomarkers

Blood biochemical data, including SCr, eGFR, hemoglobin, HbA1c, lipid profiles, liver enzymes, serum electrolytes, uric acid, and serum albumin were measured at the enrollment visit. The Modification of Diet in Renal Disease equation, which is eGFR (mL/min/1.73 m^2^) = 175 × SCr^−1.154^ × age^−0.203^ × 0.742 (if female), was used to retrieve the eGFR [33]. The first-void urine in the morning was collected within one week after the enrollment visit for urinary renal biomarkers, including urinary protein/creatinine ratio (UPCR), urinary KIM-1, urinary 8-OHdG, and urinary EGF. Urinary KIM-1, 8-OHdG, and EGF were measured using quantitative ELISA kits (Abcam, Trumpington, Cambridge, UK; ab235081 for KIM-1, ab201734 for 8-OHdG, and ab217772 for EGF). The assay types, sensitivity, assay ranges, intra-assay and inter-assay coefficients of variation were as follows: KIM-1, sandwich, 1.279 pg/mL, 7.813–500 pg/mL, 1.8–2.6%, and 1.9–6.2%; 8-OHdG, competitive, 0.59 ng/mL, 0.94–60 ng/mL, <5%, and <5%; EGF, sandwich, 1.04 pg/mL, 1.562–100 pg/mL, 8%, and 7%, respectively. To minimize measurement variation, urine samples were collected on a duplex basis at the same time by the same experienced technician. All reagents and standards for ELISA were purchased from Abcam (Trumpington, Cambridge, UK) and prepared according to standard protocols. Measurements of ELISA-based renal biomarkers were performed in adherence to standard protocols of the manufacturer, and each result was corrected using the urinary creatinine level of the same urine sample. 

### 2.4. Inductively Coupled Plasma Mass Spectrometry (ICP-MS) for Urinary Cadmium

The first-void urine in the morning was collected within one week after the enrollment visit for urinary cadmium level (UCd) measurement. Urine specimens were collected and stored in 10 mL metal-free plastic collection tubes. To avoid hydration bias, urine samples that were overdiluted or overconcentrated (urine creatinine level <0.884 or >26.520 μmol/mL) were excluded from the analysis. Urine specimens were stored at 4 °C. The UCd was quantified by ICP-MS on an Agilent 7800 ICP-MS instrument (Santa Clara, CA, USA) and analyzed using a no-gas mode. Urine specimens (500 μL) were diluted (1 + 9) with a 1.5% nitric acid (JT Baker, Phillipsburg, NJ, USA) solution containing yttrium as an internal standard. Cadmium and yttrium standards were purchased from AccuStandard (New Haven, CT, USA). The standard range was 2.67 to 355.84 nmol/L. The calibration curve had an R ≥ 0.995. The BIO-RAD Lyphochek^®^ Urine Metal Control Levels 1, 2, and 3 (Hercules, CA, USA) were used and analyzed at the beginning and end of each analytical run; then, they were analyzed again after every 10 samples. The lower limit of quantification (LOQ) for cadmium was 2.67 nmol/L. Values below the LOQ were assigned to the LOQ for analysis. Each UCd result was corrected using the urinary creatinine level of the same urine sample. 

### 2.5. Statistical Analysis

To assess the association between renal biomarkers and UCd in the CKD population, we divided the study cohort into three subclasses according to the tertiles of the UCd values. Data from the three subclasses (i.e., low, middle, and high UCd) were compared and analyzed. Categorical variables were presented as numbers with percentages and analyzed using a chi-squared test. Based on the normality of data distribution evaluated by the Kolmogorov–Smirnov method, continuous variables were presented as means with standard deviations or as medians with interquartile ranges (IQRs), and the independent *t*-test or Kruskal–Wallis H-test was used for univariate analysis. All variables with a *p*-value ≤ 0.05 in univariate analyses were assessed by multinomial logistic regression analysis to determine their associations with UCd, using the enter method and adjusting for age, smoking status, CKD staging, and diabetes. Statistical significance was set at *p* ≤ 0.05. Statistical Product and Service Solutions (SPSS) software (version 22.0; IBM, Armonk, NY, USA) was used for all analyses.

## 3. Results

### 3.1. Demographic, Clinical, and Biochemical Characteristics of Enrolled Patients

In this cross-sectional study, we enrolled 167 adult patients, including 21.56% with CKD stage 3a, 27.54% with CKD stage 3b, 29.34% with CKD stage 4, and 21.56% with CKD stage 5. The demographic, clinical, and biochemical characteristics of the study population are shown in Table 1 and Table 2 (all continuous variables are presented as median with IQR owing to non-normal distribution). The median age of the cohort was 69 years, and 32.34% of the study population were women. Of the enrolled patients, 79.04% lived in urban regions in southern Taiwan, and 12.73% of the cohort were smokers. Patients who consumed alcohol and noted betelnut usage accounted for 10.91% and 7.87% of the cohort, respectively. The most common comorbidities of the study population were hypertension (83.83%), followed by dyslipidemia (80.84%), gout or hyperuricemia (53.89%), diabetes (27.54%), vascular disease (21.56%), heart failure (10.18%), and COPD (8.98%). There were 13.17% of enrolled patients with a history of malignancy, with only a few patients having a history of transplantation (kidney transplant, 1.20%; extrarenal transplant, 1.20%). Of the enrolled patients, 40.72% received three or more types of antihypertensive agents. Additionally, 51.50% and 18.56% of the enrolled patients used lipid-lowering agents and glucose-lowering agents, respectively. The median eGFR was 28.80 mL/min/1.73 m^2^, and the median BMI was 25.04 kg/m^2^, while the median UCd was 3.43 μmol/g creatinine (IQR, 2.47–5.94 μmol/g creatinine).

### 3.2. Differences in Demographic, Clinical, and Biochemical Characteristics between Subclasses

**Figure 1:**
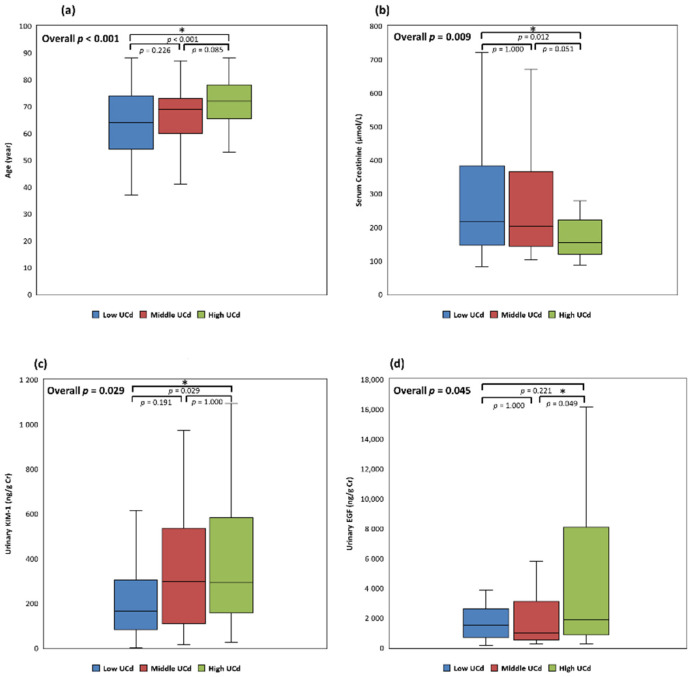
Differences in age and renal biomarkers between the three UCd subclasses. (**a**) Age between UCd subclasses; (**b**) serum creatinine between UCd subclasses; (**c**) urinary KIM-1 between UCd subclasses; (**d**) urinary EGF between UCd subclasses. *: *p* ≤ 0.05; Cr, creatinine; EGF, urinary epidermal growth factor level; KIM-1, urinary kidney injury molecule-1 level; UCd, urinary cadmium level.

To analyze the UCd-associated factors of the study cohort with low-level cadmium exposure, we divided the enrolled patients into three subclasses based on the tertiles of UCd, namely, the low UCd (≤2.71 μmol/g creatinine, *n* = 55), the middle UCd (2.72–4.62 μmol/g creatinine, *n* = 55), and the high UCd (≥4.63 μmol/g creatinine, *n* = 57) subclasses. The distribution of the demographic, clinical, and biochemical profiles in the three subclasses is provided in Table 1 and Table 2. The age of those in the high UCd subclass was greater than those in the low UCd subclass (median (IQR), 64 (54–74), 69 (60–73), and 72 (66–78) years, respectively; *p* < 0.001). In addition, the high UCd subclass had fewer patients with CKD stage 5 compared to the middle and low UCd subclasses (27.27%, 27.27%, and 10.53%, respectively, *p* = 0.044). Other demographic and clinical characteristics, such as sex, BMI, living area, drinking habits, smoking and betelnut consumption, comorbidities, as well as medications, were not significantly different between the subclasses. Furthermore, compared to the value of the low UCd subclass, the SCr value of the high UCd subclass was significantly lower (median (IQR), 215.70 (145.86–382.77), 202.44 (143.21–365.98), and 153.82 (120.22–221.88) μmol/L, respectively, *p* = 0.009) (Figure 1). Other biochemical data, including eGFR, hemoglobin, HbA1c, lipid profiles, liver enzymes, uric acid, serum albumin, and electrolytes were similar among the three subclasses. 

### 3.3. Differences in Urinary Renal Biomarkers between Subclasses

The profiles of urinary renal biomarkers were compared between the subclasses (Table 2). The urinary KIM-1 levels of the high UCd subclass were higher than those of the low UCd subclass (median (IQR), 165.75 (82.18–304.43), 295.87 (108.03–535.65), and 292.42 (156.82–583.42) ng/g creatinine, *p* = 0.029). In addition, urinary EGF levels were higher in the high UCd subclass than in the middle UCd subclass (median (IQR), 1511.25 (701.96–2604.99), 981.26 (535.40–3114.03), and 1902.74 (868.00–8079.27) ng/g creatinine, *p* = 0.045) (Figure 1). On the other hand, UPCR and urinary 8-OHdG levels were not significantly different between the subclasses. 

### 3.4. Factors and Renal Biomarkers Independently Associated with High UCd in CKD Patients

In the multinomial logistic regression analysis, after adjustment for age, smoking, diabetes, CKD staging, and covariates with a *p*-value ≤ 0.05 in univariate analyses, urinary KIM-1 level was an independent factor positively correlated with UCd in the CKD population (high vs. low UCd, odds ratio (95% confidence interval), 1.0016 (1.0001–1.0032), *p* = 0.043). Age was also positively associated with UCd after covariate adjustment (high vs. low UCd, odds ratio (95% confidence interval), 1.0534 (1.0091–1.0997), *p* = 0.018). On the other hand, the stage of CKD, SCr, and urinary EGF level were not significantly associated with UCd in the multivariate analyses (Table 3).

## 4. Discussion

In our analysis, urinary KIM-1 levels were positively correlated with UCd in the non-dialysis-dependent CKD population even after adjusting for age, smoking, diabetes, and CKD staging, which reflected the degree of renal tubular injury associated with low-level environmental cadmium exposure. Additionally, age was also an independent factor positively associated with UCd in patients with CKD. Other renal biomarkers, such as SCr, eGFR, CKD stage, UPCR, urinary 8-OHdG, and urinary EGF levels were not independently correlated with UCd in this population. The nephrotoxicity of cadmium is well known in the literature, but the ideal biomarker for cadmium-associated renal injury has not yet been defined in CKD population, especially in the low-level exposure settings. Our study focused on the patients with CKD stage 3a–5 that are considered more vulnerable to cadmium toxicity due to decreased renal clearance. Owing to the exclusion of patients with a history of occupational or accidental heavy metal poisoning at enrollment, the study cohort was composed of CKD patients who were exposed to cadmium mainly from daily sources, with the UCd of the study population being compatible with those of the general population [15,34]. Since low-level cadmium exposure, which is reflected by the UCd far below the threshold set by current guidelines, has been considered correlated with progressive renal dysfunction in the literature [35], the exact threshold of cadmium exposure associated with renal damage has been questioned, and hence, the early detection of cadmium-associated renal injury is essential in this condition. Although not statistically significant in the analyses, SCr decreased and eGFR increased with the elevation of UCd in our study regardless of the positive association between urinary KIM-1 levels and UCd. Furthermore, CKD staging and UPCR were not significantly correlated with UCd in our analyses. A similar paradox has been reported in the literature. In an analysis of UCd in lead workers, creatinine-based eGFR and creatinine clearance were positively associated with UCd, while SCr was inversely correlated with UCd [36]. Another study revealed the same phenomenon with creatinine-based renal function but not with cystatin-C-based measures [22]. Possible explanations include the time lag between tubular injury and destruction of viable nephrons, cadmium-related hyperfiltration, dependence of cadmium clearance on renal function, and effects of adjustment for urine dilution with creatinine [13,24,36]. The findings in our study support the previous observations and highlight the advantages of the novel renal biomarker-specific assessments of cadmium-associated kidney injury, which is more sensitive and especially reasonable in situations of low-level chronic cadmium exposure.

Currently, ELISA-measured kidney injury markers using urine samples have emerged as convenient and feasible tools to evaluate renal injury in various conditions, including cadmium-associated nephrotoxicity [1]. Among the novel renal biomarkers, urinary KIM-1, which is a type 1 transmembrane protein mainly produced from proximal tubule epithelial cells, has been recognized as a promising predictor of tubular injury and tubulointerstitial damage in both acute and chronic renal injury [25,37]. In animal studies of cadmium-induced renal injury, elevations of urinary KIM-1 levels were observed earlier than elevations of urinary NAG and cadmium levels after cadmium exposure. In addition, the response of urinary KIM-1 could be independent of tubular apoptosis, indicating that urinary KIM-1 might be the earliest indicator of cadmium tubulopathy [38,39,40]. In a clinical study in Thailand, urinary KIM-1 was more sensitive than urinary NAG and β2MG in detecting cadmium-associated tubular injury, which was consistent with another study in Belgium [41,42]. Research on Canadians also supported the existence of a positive correlation between UCd and urinary KIM-1 [43]. In our study, urinary KIM-1 levels measured by ELISA were positively associated with UCd in the CKD population even after adjusting for covariates of age, smoking, diabetes, and CKD staging. Despite being recognized historically in the regions with heavy cadmium pollution, overt cadmium-induced renal tubulopathy such as Fanconi syndrome is considered rare in the setting of low-level environmental cadmium exposure nowadays, and therefore, a novel and sensitive biomarker is crucial to detect and evaluate the cadmium-associated renal tubular injury in this condition [13,44]. Our analysis supports the potential use of ELISA-measured urinary KIM-1 as an early renal injury marker in low-level cadmium exposure for CKD patients, whose optimal biomarker for cadmium-induced nephrotoxicity has not yet been defined. As a sensitive biomarker for renal tubular injury, the urinary KIM-1 might also be influenced by the severity of underlying renal disease, and the urinary KIM-1 levels of this CKD population seemed relatively high compared to those of other studies in non-CKD or early-stage CKD populations [45]. However, after adjustment for covariates including CKD staging and diabetes, the independent association between urinary KIM-1 level and UCd was still identified in our study, hence indicating the role of urinary KIM-1 on the assessment of cadmium-associated renal injury in CKD patients. Furthermore, the recently developed paper-based microfluidic devices translated from conventional ELISA could facilitate the measurement of several renal biomarkers, including urinary KIM-1. With rapid and cost-effective procedures, the clinical utilization of these devices in the early detection of cadmium-associated renal injury will be a topic of interest in the future and benefit the multidisciplinary care of CKD [46,47].

Since intracellular oxidative stress is an essential part of the mechanisms of cadmium toxicity, the role of urinary 8-OHdG, a widely used oxidative stress marker [48], also has been discussed in previous studies of cadmium exposure. In a study of populations living in industrial regions of China, UCd was positively correlated with urinary 8-OHdG and urinary NAG, indicating an increase in oxidative stress associated with cadmium exposure and cadmium tubulopathy [31]. In our analysis, the urinary 8-OHdG levels measured by ELISA were not significantly different among the UCd subclasses. The differences between previous studies and our report might be partly attributed to differences in exposure levels, since our study population comprised CKD patients with low-level environmental cadmium exposure rather than people with higher exposure levels, such as those residing in industrial areas. Considering the findings of previous research and our report, urinary 8-OHdG levels might be more suitable for assessing oxidative damage in higher cadmium exposure settings. In addition, biomarkers of renal fibrosis, which is the final common result of kidney damage, have not been investigated in cadmium-associated renal injury. In previous studies, urinary EGF, a novel biomarker of renal fibrosis, was identified as an independent predictor of CKD progression, and the addition of urinary EGF to standard parameters could improve the prediction of disease events in diverse CKD populations [28]. In our study, ELISA-measured urinary EGF levels were positively associated with UCd in the univariate analysis but not in the multivariate analysis after adjusting for covariates. As a result of the chronicity and progressive nature of renal fibrosis, longitudinal investigations are warranted to evaluate the clinical efficacy of urinary EGF measurements for cadmium-associated renal injury. In summary, our study supports the role of ELISA-measured urinary KIM-1 as an early biomarker of cadmium-associated tubular injury in CKD patients with low-level cadmium exposure. Nevertheless, the potential roles of urinary 8-OHdG and EGF assessments for this condition require further study.

In our study, age was an independent factor positively correlated with UCd in patients with CKD. Similarly, epidemiologic surveys revealed that UCd increased in a stepwise fashion with age in the general population [15,49]. With a longer exposure period and slower elimination rate, the elderly are considered more vulnerable to the hazardous effects of cadmium, and our study indicates that older CKD patients should be more aware of the health influences of cadmium exposure. In contrast, the distributions of sex and smoking behavior were not different between the UCd subclasses in our study. Although smoking and female sex have been recognized as risk factors for elevated cadmium accumulation in the general population, a recent study indicated that their effects were attenuated with renal function deterioration [50,51]. The results of our analysis emphasize the importance of large-scale investigations for the specific risk factors of cadmium accumulation in the CKD population.

There were limitations to our study. This study focused on the relationship between UCd and cadmium-associated renal injury assessed by ELISA-measured urinary renal biomarkers, and blood cadmium level was not evaluated in this analysis. As another surrogate of body cadmium burden, blood cadmium level reflects a combination of both long-term and more recent cadmium exposure, whereas UCd is considered as the surrogate of long-term cadmium exposure [15,52]. Since inverse correlations between eGFR and blood cadmium levels have been found in the literature [53], further studies are warranted to assess the association between blood cadmium levels and novel renal biomarkers in CKD patients. As a result of the cross-sectional design of our study, the impact of cadmium burden on the longitudinal changes of renal biomarkers in CKD patients as well as the progression of CKD requires additional investigation. Despite the exclusion of patients with a high possibility of heavy metal exposure in our research, comprehensive evaluations of lifestyle and environmental factors could facilitate the identification of other possible confounding factors and will be essential for future studies. In addition, only Asian patients were enrolled in this study. Finally, owing to the single-center nature and the relatively small sample size of our study, the generalizability of our results could be limited by potential confounding factors, and further studies are required to define the precise threshold of UCd associated with renal injury. Despite these limitations, our findings highlight the roles of ELISA-measured urinary novel renal biomarkers in the early detection of cadmium-associated renal injury in low-level exposure settings among CKD patients, which has not been specifically studied in this population. Large-scale longitudinal investigations are needed to verify our findings and elucidate the efficacy of ELISA-measured renal biomarkers for the evaluation of progressive nephrotoxicity associated with cadmium.

## 5. Conclusions

In conclusion, our study indicated that ELISA-measured urinary KIM-1 levels were positively correlated with UCd in non-dialysis-dependent CKD patients even after adjustment for other covariates, which supports the use of urinary KIM-1 as an early renal injury marker in low-level cadmium exposure in this population. In addition, age was an independent factor positively associated with UCd in this population. Further studies are required to confirm these results.

## Figures and Tables

**Table 1:** Differences in demographic profiles and clinical characteristics between the UCd subclasses.

	Total Patients (*n* = 167)	Low UCd ≤2.71 μmol/g Cr (*n* = 55)	Middle UCd 2.72–4.62 μmol/g Cr (*n* = 55)	High UCd ≥4.63 μmol/g Cr (*n* = 57)	Univariate Analysis *p*-Value
Age (year), median (IQR)	69 (60–76)	64 (54–74)	69 (60–73)	72 (66–78) ^#^	<0.001 *
BMI (kg/m^2^), median (IQR)	25.04 (22.41–27.89)	24.46 (22.41–27.68)	25.14 (21.97–28.04)	25.22 (22.57–27.98)	0.682
Female, *n* (%)	54 (32.34)	15 (27.27)	16 (29.09)	23 (40.35)	0.275
Urban Residence, *n* (%)	132 (79.04)	46 (83.64)	40 (72.73)	46 (80.70)	0.347
CKD Stage 3a, *n* (%)	36 (21.56)	9 (16.36)	13 (23.64)	14 (24.56)	0.516
CKD Stage 3b, *n* (%)	46 (27.54)	13 (23.64)	11 (20.00)	22 (38.60)	0.065
CKD Stage 4, *n* (%)	49 (29.34)	18 (32.73)	16 (29.09)	15 (26.32)	0.757
CKD Stage 5, *n* (%)	36 (21.56)	15 (27.27)	15 (27.27)	6 (10.53) ^#,$^	0.044 *
Drinking, *n* (%)	18 (10.91)	8 (15.09)	4 (7.27)	6 (10.53)	0.425
Smoking, *n* (%)	21 (12.73)	7 (13.21)	6 (10.91)	8 (14.04)	0.877
Betelnut Usage, *n* (%)	13 (7.87)	5 (9.43)	4 (7.27)	4 (7.02)	0.877
Hypertension, *n* (%)	140 (83.83)	43 (78.18)	46 (83.64)	51 (89.47)	0.268
Diabetes Mellitus, *n* (%)	46 (27.54)	10 (18.18)	17 (30.90)	19 (33.33)	0.158
Dyslipidemia, *n* (%)	135 (80.84)	43 (78.18)	43 (78.18)	49 (85.96)	0.480
Vascular Disease, *n* (%)	36 (21.56)	9 (16.36)	11 (20.00)	16 (28.07)	0.303
Heart Failure, *n* (%)	17 (10.18)	3 (5.45)	7 (12.72)	7 (12.28)	0.366
COPD, *n* (%)	15 (8.98)	4 (7.27)	6 (10.91)	5 (8.77)	0.799
Gout or Hyperuricemia, *n* (%)	90 (53.89)	29 (52.73)	31 (56.36)	30 (52.63)	0.904
Kidney Transplant, *n* (%)	2 (1.20)	0 (0.00)	1 (1.82)	1 (1.75)	0.608
Extrarenal Transplant, *n* (%)	2 (1.20)	1 (1.82)	0 (0.00)	1 (1.75)	0.608
Previous Malignancy, *n* (%)	22 (13.17)	7 (12.73)	5 (9.09)	10 (17.54)	0.414
Lipid-Lowering Agent, *n* (%)	86 (51.50)	26 (47.27)	30 (54.55)	30 (52.63)	0.731
Glucose-Lowering Agent, *n* (%)	31 (18.56)	6 (10.91)	13 (23.64)	12 (21.05)	0.192
Antihypertensives ≥ 3 types, *n* (%)	68 (40.72)	27 (49.09)	19 (34.55)	22 (38.60)	0.276

*: *p* ≤ 0.05; ^#, $^: significantly different compared to the low or to the middle UCd subclass, respectively. BMI, body mass index; CKD, chronic kidney disease; COPD, chronic obstructive pulmonary disease; Cr, creatinine; IQR, interquartile range; *n*, number; UCd, urinary cadmium level.

**Table 2:** Differences in biochemical profiles and renal biomarkers between the UCd subclasses.

	Total Patients (*n* = 167)	Low UCd ≤2.71 μmol/g Cr (*n* = 55)	Middle UCd 2.72–4.62 μmol/g Cr (*n* = 55)	High UCd ≥4.63 μmol/g Cr (*n* = 57)	Univariate Analysis *p*-Value
**Biochemical Profiles, Median (IQR)**					
Serum Creatinine (μmol/L)	177.68 (129.95–288.18)	215.70 (145.86–382.77)	202.44 (143.21–365.98)	153.82 (120.22–221.88) ^#^	0.009 *
eGFR (mL/min/1.73 m^2^)	28.80 (16.20–42.00)	25.80 (13.20–39.00)	28.20 (13.20–42.00)	37.20 (24.00–43.80)	0.076
Hemoglobin (g/L)	120.00 (105.00–131.00)	120.00 (106.00–131.00)	120.00 (105.80–135.00)	114.00 (101.00–130.00)	0.626
Glycated Hemoglobin (%)	5.90 (5.50–6.30)	5.65 (5.40–6.10)	5.90 (5.55–6.45)	5.90 (5.70–6.33)	0.235
Total Cholesterol (mmol/L)	4.27 (3.76–4.89)	4.22 (3.78–5.05)	4.35 (3.72–4.88)	4.11 (3.53–4.70)	0.474
LDL-C (mmol/L)	2.39 (1.90–2.82)	2.40 (2.02–2.92)	2.42 (1.90–2.83)	2.28 (1.74–2.80)	0.634
HDL-C (mmol/L)	1.22 (1.01–1.53)	1.24 (1.04–1.53)	1.22 (1.01–1.57)	1.18 (1.01–1.50)	0.788
Triglyceride (mmol/L)	1.38 (0.94–1.85)	1.33 (0.77–1.77)	1.54 (0.98–1.98)	1.34 (1.00–1.84)	0.449
ALT (μkat/L)	0.28 (0.20–0.42)	0.31 (0.20–0.47)	0.28 (0.21–0.34)	0.28 (0.20–0.42)	0.728
Albumin (g/L)	43.60 (41.30–45.80)	44.00 (40.80–45.80)	43.70 (42.30–46.00)	42.60 (41.10–45.50)	0.295
Uric Acid (mmol/L)	0.37 (0.31–0.43)	0.37 (0.30–0.43)	0.36 (0.31–0.43)	0.37 (0.30–0.43)	0.987
Ca (mmol/L)	2.30 (2.24–2.38)	2.30 (2.20–2.38)	2.30 (2.24–2.40)	2.33 (2.25–2.40)	0.548
P (mmol/L)	1.20 (1.08–1.36)	1.23 (1.07–1.42)	1.23 (1.08–1.36)	1.16 (1.08–1.32)	0.464
K (mmol/L)	4.50 (4.10–4.80)	4.50 (4.10–4.80)	4.50 (4.10–4.73)	4.40 (4.15–4.80)	0.939
**Urinary Renal Biomarkers, Median (IQR)**					
UPCR (mg/mmol Cr)	59.21 (15.06–128.14)	82.33 (29.97–161.92)	38.97 (12.15–176.32)	44.76 (10.88–109.80)	0.124
KIM-1 (ng/g Cr)	253.29 (117.52–506.29)	165.75 (82.18–304.43)	295.87 (108.03–535.65)	292.42 (156.82–583.42) ^#^	0.029 *
8-OHdG (μg/g Cr)	93.93 (51.36–205.27)	87.35 (54.84–223.24)	108.51 (58.81–177.04)	80.39 (47.12–220.06)	0.921
EGF (ng/g Cr)	1447.83 (700.08–3681.40)	1511.25 (701.96–2604.99)	981.26 (535.40–3114.03)	1902.74 (868.00–8079.27) ^$^	0.045 *

*: *p* ≤ 0.05; ^#^: significantly different compared to the low UCd subclass; ^$^: significantly different compared to the middle UCd subclass. 8-OHdG, urinary 8-hydroxy-2-deoxyguanosine level; ALT, alanine aminotransferase; Ca, blood calcium level; Cr, creatinine; EGF, urinary epidermal growth factor level; eGFR, estimated glomerular filtration rate; HDL, high-density lipoprotein cholesterol; IQR, interquartile range; K, blood potassium level; KIM-1, urinary kidney injury molecule-1 level; LDL, low-density lipoprotein cholesterol; *n*, number; P, blood phosphorus level; UCd, urinary cadmium level; UPCR, urinary protein/creatinine ratio.

**Table 3:** Factors and renal biomarkers independently associated with UCd in multivariate analysis.

	Middle UCd vs. Low UCd		High UCd vs. Low UCd	
	Odds Ratio (95% CI)	*p*-Value	Odds Ratio (95% CI)	*p*-Value
Age (year)	1.0229 (0.9862–1.0610)	0.224	1.0534 (1.0091–1.0997)	0.018 *
SCr (μmol/L)	1.0004 (0.9973–1.0036)	0.796	1.0005 (0.9972–1.0039)	0.751
KIM-1 (ng/g Cr)	1.0010 (0.9994–1.0025)	0.221	1.0016 (1.0001–1.0032)	0.043 *
EGF (ng/g Cr)	1.0000 (0.9999–1.0001)	0.653	1.0000 (1.0000–1.0001)	0.262
Diabetes	1.8685 (0.6577–5.3080)	0.241	2.2529 (0.7447–6.8151)	0.150
Smoking	1.2985 (0.2712–6.2168)	0.744	0.8577 (0.1484–4.9573)	0.864
CKD Stage 3a	Reference		Reference	
Stage 3b	0.6638 (0.1678–2.6268)	0.559	1.2347 (0.2973–5.1276)	0.772
Stage 4	0.5730 (0.1395–2.3543)	0.440	0.5822 (0.1255–2.7015)	0.492
Stage 5	0.4929 (0.0784–3.1010)	0.451	0.2050 (0.0250–1.6808)	0.140

*: *p* ≤ 0.05. CI, confidence interval; CKD, chronic kidney disease; Cr, creatinine; EGF, urinary epidermal growth factor level. KIM-1, urinary kidney injury molecule-1 level; SCr, serum creatinine; UCd, urinary cadmium level.

## Data Availability

All the data generated in this study are available from the corresponding author (leewenchin@gmail.com) upon reasonable request due to research regulation of the hospital.
